# The Lectures of Sir Astley Cooper, Bart. on the Principles and Practice of Surgery, with Additional Notes and Cases

**Published:** 1826-01

**Authors:** 


### VIII.

*The Lectures of Sir Astley Cooper, Bart. F.R.S. Surgeon to the King, &c. on the Principles and Practice of Surgery; with additional Notes and Cases.* By FRED. TYRRELL, Esq. Surgeon to St. Thomas's Hospital, and to the London Ophthalmic Infirmary. Vol 1. pp. 352. 1824.

LECTURES on surgery, like those on chemistry, lose a very great proportion of their value and interest, when transferred from the lecture-room to the printing-office. The anatomical and pathological specimens, together with the minute colloquial elucidations, constitute so important a feature in a course of surgery, that very few lectures of this kind will bear publication, without certain detriment to the lecturer. A recent and memorable example of this is so fresh in the minds of all, that we have no occasion to specify names. With all these drawbacks, the lectures of Sir Astley Cooper excited an intensity of interest, and attained an extent of circulation on their first ap-

"* Three, four, and even five scruples of calomel were usually taken, before this effect was produced; and the black, grey colour seemed always indicative of the action of calomel, being precisely the colour which is produced by calomel combined with ammonia."

pearance in the Lancet, quite unparalleled in the annals of medical stenography. The speculation succeeded so well indeed, that another eminent surgeon was quickly brought on the tapis; but it was soon discovered, to the *cost* of more than one party, that—"non ex aliquovis ligno fit Mercurius." It was found that what was a monstrous good *vivâ voce* joke in the class-room, or even in the Court of Chancery, was a monstrous bad one in a medical publication!—in short, the thing turned out, in the end, to be *no joke* at all. This being the case, to what are we to attribute the interest excited by our present author's lectures? We conceive that this interest is partly owing to the reputation of the lecturer, and the deep conviction in the minds of his brethren, that no other surgeon in this kingdom has ever had such an unbounded field of observation and experience. But although the interest might have been *excited* in this way, it could not have been *maintained* as it has been, without some other cause. What this other cause is, no man of common apprehension can be long in discovering. It is the rich tissue of authentic facts and legitimate deductions which spreads itself over every page in the work, unsullied by any predominating theory or hypothesis which, like a vapoury atmosphere, distorts every object seen through it. Something also may be attributable to the freshness of the facts and frankness in the manner of stating them, which are far more prepossessing than studied elegance of language and refinement of thought—too often the substitutes for fertility of materials. The ample experience of Sir Astley Cooper has enabled him, on all occasions, to illustrate his subject with the most striking examples, while the tone of urbanity and good feeling which pervades all his allusions to the character and practice of his brethren, forms a striking contrast with that censorious and even vituperative spirit which too often pervades the lectures and writings of modern medical men, who seem not to *reflect*, that in injuring others they *doubly* injure themselves.

——————— how few
Know their own good, and knowing it pursue!

It is hardly necessary to observe, that Sir Astley's lectures have been extensively read through the medium of the Lancet; but after granting all that can be demanded for the degree of accuracy to which stenographers have arrived, it must be evident that, in taking down medical lectures, the non-professional short-hand-writer is liable to commit many errors both of conception and notation. It was, therefore, natural and proper that Sir Astley should wish to see a correct copy of his lectures diffused among his brethren, and this task has been performed by his relative Mr. Tyrrell, each sheet having been corrected by the lecturer himself. This fact, authenticated by a letter from Sir Astley Cooper to the author, is a sufficient guarantee forthe correctness of the copy under review, and must ever render it the proper standard of reference when this pre-eminent surgeon's lectures or opinions are quoted in future.

A course of lectures is like an elementary work—it is not adapted for a regular analysis. Such course must contain the rudiments, as well as the *illustrations* of the science taught. It is with the *latter*, and with the *peculiar opinions* of the lecturer that the Journalist can have any thing to do—and of *these* we propose to take a rapid *coup d'oeil*, in this and in a few succeeding numbers of our Journal, convinced that our gleanings will afford the reader wholesome food for reflection, as well as useful materials for direct application to the daily concerns of his avocation.

The first volume contains twelve lectures, and embraces several important subjects, including irritation, inflammation, suppuration, ulceration, gangrene, injuries of the head, &c.—We shall glance at these in order laid down.

I. *Irritation.* The study of this phenomenon is considered by Sir Astley as the foundation of medical science. The doctrine of irritation teaches the immediate and remote effects of injuries, and is, in fact, a subject of immense extent, both in surgery and the practice of physic. In the outset, Sir Astley takes occasion to allude to *sympathy*, an expression which it is fashionable among sciolists and closet practitioners to laugh at, as a term used to cloak our ignorance. But the same might be said of many other doctrines and terms in our art. For instance, every one knows what is meant by *irritation*, yet no one can explain the rationale of it. We see the irritation of a splinter produce tetanus, but who can tell us the *modus agendi?*—So we find that if cold water is thrown on the lower extremities a contraction of the muscular fibres of the intestines takes place—and we say it is by *sympathy*. But because we cannot explain *how* this takes place, the term sympathy is laughed at. It is not ridiculed, however, by the eminent author under review.

"The real nature of sympathy is yet unknown, but we are acquainted with many of its effects; and, as an example, we may give the communication which exists between the uterus and breasts; as the former is impregnated, and the various changes proceed in it during the period of gestation; so corresponding alterations are proceeding in the breasts, the glands enlarge gradually, the nipple becomes elongated, and the secretion of milk commences; thus, before the child is born, nature has carefully provided for its future support. Many other of the natural functions of the body are supported upon the same principle; as sneezing, which is a sympathetic action between the nose, velum palati, and the abdominal muscles, instituted to remove causes of irritation from the nose: coughing, which is a sympathy between the larynx and abdominal muscles: breathing and the expulsion of the fæces are also sympathetic functions, and with these a multitude of other examples might be given." 3.

It is doubtless through the agency of the nerves that most of the phenomena of sympathy are produced, and many ingenious explanations have been given of individual instances, by Mr. Bell and other physiologists, but there are numerous other instances where the connexion of nerves gives but a very unsatisfactory solution of the question.

"In some instances," says Sir Astley, "the course of irritation is from the irritated part to the sentient extremity of the nerve, as the pain experienced in the knee and foot from a disease of the hip; or the pain in the little finger and half of the ring finger, when the ulnar nerve is struck at the elbow: injuries of the brain produce vomiting, their influence being imparted to the stomach through the medium of the eighth pair of nerves. In other cases, the course of sympathy is from the affected part to the origin of the nerve; thus pain in the loins is consequent on diseased testicle; or pain between the shoulders from affection of the liver. Occasionally the sympathetic communication is through the brain, as the following case will prove. Mr. Toulmin, of Hackney, attended a lady on account of her suffering severely from a diseased tooth, and she appeared also to be afflicted with hemiplegia. Mr. Toulmin extracted the tooth by the lady's desire, and in a short time the paralytic affection entirely subsided." 6.

Several other cases of this kind are mentioned by our author. Thus, two persons came from the same town, and on the same day to consult him, each with an opening in the cheek, communicating with abscess near the alveolar process. These openings were of long standing. He directed a diseased tooth to be extracted near each ulcer, and they both quickly healed. A lady had been long afflicted with a fungoid granulation protruding through an opening in the cheek. It had resisted every remedy, till a tooth nearly opposite the opening was withdrawn, and there was no longer any difficulty in curing the fungoid granulation. A gentleman had an ulcer of long standing on his chin, which could not be healed. At length a tooth became painful, and was extracted. The ulcer immediately healed. These cases are mentioned to shew the necessity of diligently seeking the causes of irritation.

Of the *general effects* resulting from local irritation, Sir Astley instances what occasionally takes place after introducing a bougie into the urethra, viz. sickness, pallor, faintness, and sometimes a single paroxysm of ague.

At the time of writing this, an example of sympathetic urethral irritation is under the care of Mr. Thomas, of Leicester place, and the writer of this article, which, we believe, has not a parallel in the annals of medicine. A young gentleman, Mr. L——, about 27 years of age, had a bougie passed at two p. m. on Tuesday, the 6th of September. At six o'clock in the evening a sense of coldness and slight nausea came on, but did not amount to actual faintness. He took a dose of salts of his own accord, which acted on his bowels, and produced vomiting, but he passed an uncomfortable night. All day on Wednesday the patient was ill, but was not seen by any medical man. On Thursday morning Mr. Thomas was called to him, who found him in a very strange condition. No pulse could be felt. The extremities were cold—the fingers blue—the breathing short, and rather laborious; yet the patient was sensible, though somewhat deaf, and confused in his ideas. Some diffusible stimuli were exhibited, and on Thursday night at ten o'clock, the writer of this article joined Mr. Thomas in consultation. A very particular examination was now made of the arterial system. A weak fluttering, and indistinct movement was felt in the region of the heart, when the patient lay over on that side—the chest sounded badly in every part—no respiratory murmur could be heard through the stethoscope, but the situation was unfavourable for auscultation, being in one of the most noisy streets of the Metropolis—no pulsation could be felt in the descending aorta, the inguinals, carotids, radials, or, in short, in any artery of the body. The examination was repeatedly made, and with the utmost care. The patient was sensible, but restless, and complained of an indescribable oppression, and sense of anxiety in his chest. He was thirsty—the tongue dry and furred—the countenance clouded—the extremities cold and pale—the nails blue—the breathing very short and somewhat laborious. Bottles of hot water were applied to the extremities, and diffusible stimuli of the most powerful kind were exhibited internally, though with some difficulty, as the stomach was irritable. In this condition he remained the whole of Thursday night, and was found on Friday morning exhibiting precisely the same phenomena. The stimulation with frictions, fomentations, &c. &c. were continued. At 5 p. m. the medical attendants again met, but not the slightest mark of arterial action could be felt in any tangible artery. He had not slept any in the night, or during the day. The tongue was more loaded—there was some intellectual aberration, and the muscular debility was extreme. He could hardly turn himself in bed, and when his head was raised, he was threatened with syncope. A stimulating salt-water bath was ordered at ten o'clock at night, and in the mean time the stimulation was continued. This day he took 20 grains of calomel, with compound extract of colocynth, and the bowels were well cleared. At half-past ten at night, while preparing to place the patient in the warm bath, a slight pulsatory motion could be felt in the carotids, and shortly afterwards, while in the bath, the radial arteries began to pulsate, though very feebly. From this time a gradual and almost imperceptible improvement took place in the arterial action and animal temperature, till both were completely developed. The freedom of breathing corresponded with the improvement in the function of the circulation, and, in two days from the re-appearance of arterial pulsation, a smart febrile reaction was set up, which required moderate depletion.

After this, a series of phenomena presented themselves, which were as puzzling in respect to their etiology as they were difficult in a therapeutical point of view. Symptoms of inflammation in the whole line of the alimentary canal came on, from the mouth to the anus, with an exanthematous eruption over the whole body. And last of all, a hard swelling was found in the perineum, in the site of the stricture, causing great difficulty in voiding his urine, with paroxysms of cold, hot, and sweating stages of fever, indicating the formation of matter. It was conjectured at one time, but never proved, that the patient had taken some deleterious substance in conjunction with, or in a mistake for, the dose of Epsom salts, at the beginning. This, however, has since been nullified.

After several days of vacillation, during which he sometimes gave hopes of complete recovery, and at others presented alarming symptoms, nothing decisive occurred. At length, on the 26th September, the scrotum suddenly swelled, and exhibited obscure fluctuation. But when freely incised, no matter could be found. The abdomen now became tense, swelled, and painful, with incessant vomiting. A blush of inflammation could be seen running up from the testicle of the left side to the iliac region, with great tenderness to the touch. On the 27th, when the abdomen was amazingly distended, and the pulse 144, with constant vomiting, all pain suddenly ceased, and the patient appeared dying. At this time a very large enema was thrown up, which brought away a large quantity of thin feculent matter, the patient having taken several doses of calomel previously. The vomiting ceased—the tension of the abdomen abated—and an apparently phlegmonous inflammation was established all along the left side of the abdomen, from the perineum to near the false ribs. Large poultices and fomentations were assiduously applied, and the suppuration appeared to advance rapidly--the constitutional symptoms abating considerably. On the evening of the 29th September the apparent abscess was very prominent, and a fluctuation could be felt. Some little doubt, however, was thrown over the precise nature of the contents, in consequence of an emphysematous feel which the tumour communicated. At all events, it was deemed proper to open it: but when a lancet was pushed in, nothing but air rushed out, and the tumour sunk to a level with the rest of the abdomen! Here was one more puzzling phenomenon added to the many which presented themselves in the course of this most mysterious case. An opiate being given, he slept a good deal this night. 30th. There was a good deal of watery discharge mixed with air from the wound, and also from the original wound in the scrotum. At length a profuse suppuration was established in the cellular membrane, between the muscles and integuments of the abdomen, under which he nearly sunk, but was ultimately saved by extensive openings, bark, acids, and nourishing diet.

When the suppuration was nearly drained off, the swelling subsided, and the patient rapidly recovering his health, it was found that a proportion of urine issued from a small opening in the urethra, near the seat of the stricture, and afterwards from the incisions in the side. Thus, in fact, there can be no doubt that the urethra was slightly ruptured by the bougie in the first instance, and that the diffuse inflammation in the scrotum, groin, and side of the abdomen was the result of some small extravasation of urine. To this source of irritation, therefore, we must attribute the whole series of phenomena in this most remarkable case.

We do not recollect any case on record where there was a similar cessation of arterial action in all tangible arteries for so long a period, and with ultimate recovery. A case bearing some analogy to it was published, several years ago, by Drs. Johnson and Lara, where a person had no perceptible action of the arteries for about 36 hours; but the patient was old, and had thoracic dropsy. He died suddenly some weeks afterwards. The present case we believe to be unique in its way, and highly illustrative of the sympathy which exists between different systems in the animal economy. Whatever was the source of sympathetic irritation in this case, (but there is no doubt of its being in the urethra) it is evident that there was no defect in the heart or arteries to account for the phenomena abovementioned. Their *functions* must have been disturbed and interrupted from sympathy with some other part.

But to return to our author. Sir Astley illustrates the *general effects* of local irritation or injury by a variety of examples, as by the destruction of life from a slight blow on the stomach, nothing being cognizable on dissection. Such fatal affections are, we should conceive, from the shock given to the semilunar ganglion, and the various important nerves radiating from that sensorium abdominale.

Constitutional irritation from local injury is too often and too fatally exemplified in compound fractures of the legs. Pain in the loins, extending up the spine to the head—restlessness—anxiety—furred tongue—loss of appetite—nausea and vomiting—deranged or suspended secretion of bile, urine, perspiration, &c. evince the affection of the nervous and digestive organs—while the participation of the circulating system is sufficiently proved by the hard, irregular, and ultimately intermitting pulse. Finally, the sensorial functions are greatly disturbed, and the patient is worn out by irritative fever, unless the—

————————Immedicabile vulnus

Ense recidendum!

The following is Sir Astley Cooper's rationale of the foregoing train of phenomena. Some parts of the explanation are open to objections, and others, though correct in principle, are rather fancifully expressed—but, upon the whole, the rationale is as good as can be offered in the present state of our knowledge.

"Thus in constitutional irritation, whether from injury, or from external or internal disease, every part of the system may be affected, and it appears to take place in the following way: When a part of the body receives an injury, the nerves convey a knowledge of it to the important organs, as the spinal marrow, brain, heart, stomach, &c.: nature immediately commences the restorative process, by stopping all the customary secretions; the various outlets being thus closed, the blood collects in quantities in the heart and large blood vessels, which propel it with unusual force to the injured part; giving rise to inflammation in whatever form can best accomplish the desired effect. This is an illustration of the manner in which nature contends for a cure; she occasionally requires to have her ardour checked, or aided, in proportion to her powers: we must watch with 'eagle's eyes' her proceedings, and be exceedingly cautious in our interference: for by restoring the natural secretions too soon, we may, by thus abstracting blood from the injured part, prevent the restorative process; or, by adding to excitement, we may prevent the beautiful and judicious operations of nature, by producing too much action." 13.

Our author observes that excessive irritation frequently follows operations on very young subjects, but rarely on those performed on people of advanced age. He recommends that lithotomy should not be performed on children under two years. Persons deprived of their natural rest, and who take little food, suffer more from injuries than those under opposite circumstances. The same may be said of intemperance and sobriety. The necessity of informing ourselves of the habits of patients before operating on them, is sufficiently exemplified by the following case of Mr. Tyrrell's, published in a note.

*Case.* " John Westrip, æt. 30, was admitted into St. Thomas's Hospital on account of a severe injury to the elbow joint, caused by the wheels of a loaded coal waggon passing over the part. The nature and extent of injury were such, that I thought it advisable to amputate the limb. This was done about twenty hours after his admission into the hospital, as he would not submit to have it performed sooner. He was much intoxicated when the accident happened, but I could not get any information as to his previous history from the persons who came with him, for they had not been before acquainted with him. On the second day after the operation he had severe constitutional suffering; his pulse was very quick, his skin hot, his countenance anxious; the stump was much inflamed, and very painful: these symptoms rapidly increased, he became delirious, and the edges of the wound began to slough; the usual remedies were given to subdue the irritation, but without producing relief. On the evening of the day after he became delirious, I learnt from one of his friends who came to see him, that he had been in the habit of taking ten or twelve pints of porter daily, besides spirits. I immediately sent for some porter, which he drank with great eagerness; and in the course of an hour after, he fell asleep. He slept several hours, and awoke perfectly composed and sane: the porter was continued daily, with a few ounces of wine; the sloughing of the stump stopped, and he rapidly recovered." 16.

In this place Sir Astley Cooper, and also Mr. Tyrrell, touch on the subject of dissection-wounds, and both seem to be of opinion that the bad consequences which occasionally result, are more frequently dependent on the state of the constitution at the time, than on any morbid poison introduced. We agree so far with Sir Astley and his editor, as to think that a great deal depends on the state of the constitution; but this does not do away with the existence of a morbid poison introduced. Of two men exposed to the poison of typhus, or the miasma of a dangerous marsh, one will become affected from the state of predisposition, while the other, from high health, will escape. But would any one argue from this, that no contagion existed in typhus or miasmata in a marsh? Again. How is it that dissection wounds, received while examining women who die of *puerperal fever*, are so often productive of injury, not only in the predisposed, but in the most healthy dissectors? We aver, from observation and examination of the records of such cases, that almost all the injuries which happen, are in dissections of subjects who have had *inflammations* of one kind or other at the time of death. Can this be accidental? No, assuredly. The denyers of a morbid poison, or of the absorption of putrid animal matter, ask, " how is it that it is principally towards the close of the season, when the students are out of health, that we see the bad effects of dissection-wounds?" We answer that, unquestionably they are then more susceptible of the poison than at the beginning of the season, when they are fresh from the country, and not debilitated by hard study and bad air. But we, in turn, beg to ask two questions. How is it that we very often see men in the prime of life, and in country practice, affected by these dissection-wounds?—And how is it that, among the squalid wretches and debilitated drunken mechanics of this metropolis, who are every day and every hour receiving punctured wounds from various instruments, we rarely hear of any serious consequences as the result? Surely if nothing depended on a morbid poison or putrid matter, and every thing depended on constitution, we should as often see bad consequences from punctured wounds among mechanics, as from dissection-wounds among students. But the case is quite the reverse. Our creed then is, that in certain dead bodies, and in certain states of dead bodies, there exists a morbid poison or a putrid matter, (call it what you please) which is capable of occasionally affecting the strongest constitutions when introduced by a wound, but which produces its effects infinitely more often and readily in those constitutions which are out of health, than in those under opposite circumstances. We do not make these remarks to prevent attention to the constitution, but to prevent the whole attention being directed there, and no importance attached to the prevention of the evil consequences—namely, the prevention of the introduction and absorption of morbid poison. If the existence of this lastmentioned agent comes to be denied in toto, no care need be employed in dissections by those who are in good health. But this view of the case is, we are convinced, a very dangerous one.

It is hardly necessary to say, that our able author lays down admirable rules for the treatment of irritation, local and general; we can make room for only one extract, which, however, comprises a great deal of judicious advice and practical knowledge.

"There are two means of reducing irritation.

"First, by restoring the secretions of the different organs, and, by thus opening the outlets, lessen fever. A man who has his skin hot and dry, feeling excessively heated, if you can produce a free perspiration, will be immediately relieved and become cool. When the irritation is severe, you must not limit your medicine to act on any particular organ, but try to restore all the secretions; and this is best effected by administering mercurials to act on the liver, aperients on the intestines, diuretics on the kidneys, and antimonials on the skin.

"The second mode of relieving irritation is by allaying the excitement of the nervous system; this can be effected by giving opium and antimony combined; or calomel, antimony, and opium, to act on the skin or liver as well as the nervous system: the latter is one of the best medicines for allaying irritation, and may be given to adults in doses of two grains of calomel, two of antimonial powder, and one grain of opium: to this you may add saline medicines, for they promote the secretions and lessen the irritability of the nervous system. Liquor ammoniæ acetatis with tinctura opii, and the common saline with opium, soothe the system into peace. The alkalies, as potash and soda, diminish the irritable actions of organs, as may be seen in irritable bladder. Hyoscyamus and conium are also excellent remedies, especially in those persons with whom opium disagrees.

"The abstraction of blood lessens the momentum of the circulation, and prevents the danger of congestion in any of the vital organs; but it must be taken away with the greatest care, not to diminish too much the powers of the constitution. A man was taken into Guy's Hospital, having a concussion on the brain; the dresser, who admitted him, was a great admirer of venesection, and consequently bled the patient frequently, and in large quantities; in ten days the man died. On examining the head after death, a very slight laceration of the brain was discovered, but no attempt at restoration: the continued abstraction of blood had deprived nature of her restorative powers. In compound fractures it is extremely dangerous to bleed largely; as, by lessening the power of the constitution too much, there is not sufficient energy to perform the task of reparation.

"If an important disease exist, nature will not always have power to perform the necessary duty of restoration. A man was admitted into St. Thomas's Hospital, under Mr. Cline, for a simple fracture of the os humeri; the fracture did not unite, and scarcely any inflammation arose: on the twenty-ninth day the man died suddenly. Upon dissection an aneurism was found in his aorta, which had burst: very little if any change had taken place in the fractured part.

"When there is chronic irritation, you can only restore the system to healthy action by slowly acting on the secretions; to produce these diseases, some slow feverish action has existed, and some one of the secretions has been lessened; the skin is dry, or the bowels are costive; the bile is not properly secreted, or the urine is less abundant; hence the blood is locked in the system, and congestion, followed by inflammation, produces local diseases. The pil. hydrarg. submur. comp. is the best remedy under these circumstances, as it increases the secretions of the liver, intestines, kidneys, and skin. The blue pill, or calomel, should be followed by an aperient in the morning, as they act on the liver, but not in proportion on the other secretions. To attempt to cure such diseases suddenly, or by violent and active means, must be ever improper; a chronic treatment is required, and by slow degrees only can you restore the body to a healthy state. Let me repeat, *all the secretions must be restored, as this is the grand principle in the cure of disease.*" 29.

Under the head of "influence of the mind upon the body," Sir Astley Cooper makes many remarks of a highly interesting nature, not only as regards the patient, but the practitioner also. It is, indeed, astonishing what a difference the temper and disposition of a man make in the chance of recovery from an accident or a disease. Much of the prognosis may be founded on the appearance of a patient, even in the first few hours after a severe injury. If he submit himself to his fate without repining—if he readily yield to the advice of his friends, and consent to every thing that is proposed for his relief, he generally does well. If on the contrary, he bitterly laments his fate—is too anxious about the means of cure; and impatient in their not being immediately obtained, there is then a constitutional irritation highly unfavourable to recovery.

"It is the surgeon's duty to tranquillize the temper, to beget cheerfulness, and to impart confidence of recovery. Some medical practitioners are so cold and cheerless as to damp every hope; whilst others inspire confidence of recovery, and a disregard of situation, which supports the regular performance of all the actions necessary for restoration. It is your duty, therefore, to support hope, to preserve tranquillity, and to inspire cheerfulness, even when you are still doubtful of the issue." 31.

Grief has a peculiar and powerful effect in lowering the actions of the body—arresting the secretions, particularly of the liver—and producing a low feverish state. The two worst forms of disease to which the human body is liable, cancer and fungus, are frequently, in Sir Astley's opinion, induced by grief and anxiety. Anger, too, has the power of greatly disturbing the healthy actions of the body, and retarding the progress of recovery.

But *fear* has the greatest influence of all other emotions in destroying our best efforts to cure injuries or diseases. Often do patients declare, under such circumstances, that they cannot recover—and this prepossession seems to deprive their constitutions of all restorative power. A patient applied to Sir Astley, not long since, for symptoms that indicated stone in the bladder. He sounded, and touched the stone. On informing the patient of this, the latter replied, "I hope this is not the case, for I can never submit to an operation." The patient returned into the country, and died in a few days afterwards! Several other curious instances of the influence of fear are given from the extensive experience of our author, and the lecture on irritation concludes.

II. *Inflammation.* This extensive—we might almost say universal subject, is treated by Sir Astley Cooper with all that talent and discrimination for which he is so remarkable. We can merely glance at some of the most curious and original portions of it.

Speaking of the different kinds of inflammation, our author introduces the following original observations.

"There is another kind of inflammation, which I would call the irritable; in this disorder the blood-vessels are much less affected than the nerves. You are called to attend a person, who tells you that he feels in the hand, arm, or some other part, most agonizing pain: if not experienced in these matters, you will be inclined to doubt the correctness of your patient's statement, as you cannot discover any diseased appearance of the part. Some time ago I was consulted by a lady who had this painful affection in the foot, and I employed various remedies without her being relieved: finding no improvement, and suffering in health, she went to the coast, and there used a steam-bath; and, without any further means, the pain quickly subsided. The eyes are very subject to this torturing disorder. But no part is more frequently attacked by it than the breasts of young women. It produces such a degree of tenderness, that they cannot bear the slightest pressure: the pain extends to the shoulder, down the arm, and even to the elbow and fingers, accompanied by constitutional irritation. To cure these pains, and general derangement, such medicines must be given as will influence the secretions, but especially that of the uterus. This irritable inflammation sometimes attacks the testicles, rendering them extremely sensitive, so that the patient can scarcely sustain the pressure of his clothes. It is attended with little increase in the size of the gland. I have been obliged to remove the testicle in three persons for this disease. The subject of one of these operations was a gentleman from South Carolina: he came to England for advice, and consulted many of the surgeons in London, but without experiencing any relief of his sufferings, from the various remedies they advised. He at length requested me to remove the torturing part; this I did, and he returned to his native country quite well. The bladder is also occasionally disordered by this irritable inflammation, and the symptoms, in many respects, resemble those of stone; in both cases there is pain in making water, and blood is sometimes mixed with the urine. The grand difference in these two cases is this: the irritable bladder is most painful when distended; that which contains a stone, when emptied. On dissection, the inner coat of an irritable bladder has been seen the colour of red velvet. I have known this irritable inflammation attack the rectum, and produce excessive suffering, which was relieved by large doses of soda.—Soda, rhubarb, and the compound powder of ipecacuanha, are the best remedies." 54.

Although our author has made experiments on animals with the view of ascertaining the pathological condition of inflamed parts, yet he wisely avoids going farther into the theory of inflammation than drawing such conclusions as are obvious from the evidence of the senses. He then passes on to a more important subject, the treatment. Here, as usual, the man of practical observation and wide experience appears at every step. Upon our paramount remedial agent, blood-letting, Sir Astley makes many highly judicious remarks. The state of the pulse and the appearances of the blood daily lead practitioners into error. *Hardness* of the pulse is a far better indication for bleeding than *quickness*. The latter, in fact, is in itself, no proof that bleeding is necessary. " If hardness and quickness are united, no additional evidence of its necessity can be wanted." We doubt whether there are not exceptions even to this rule, general as it is. We are at this time attending an elderly gentleman, to whom we were called a few days ago. His pulse vibrated like a rod of iron and was very quick, considering his age. But the other phenomena of inflammation were wanting, and we did not bleed. An aperient medicine and an enema brought away some very indurated fæces, and immediately the hardness and quickness of the pulse vanished. Here then the condition of the circulation depended on irritation in the bowels and bleeding was, to say the least, unnecessary.—The appearance of the blood is still more deceitful.

" The indication for a repetition of bleeding is said to be a buffy state of the blood; but your decision must not be governed by this appearance alone, for you must still have regard to the pulse.

" When blood is cupped or hollowed upon its surface, it is said to be a proof of inflammation, and that bleeding should be repeated; but the following case will show, that even a cupped state of the blood, and buff conjoined with it, are not sufficient evidence that venesection may be repeated. A patient in Guy's Hospital, in the last stage of scurvy, whose blood vessels were so weak, that a slight pressure on the skin produced ecchymosis, whose gums frequently bled, and whose pulse was exceedingly quick and feeble, had a very small quantity of blood taken from his arm as an experiment; after standing for a few hours, it became not only buffy, but considerably cupped." 62.

Many excellent directions for the abstraction of blood are laid down by the lecturer; and then he points out the second best mean of controlling inflammation, namely an increase, or rather a restoration of the secretions, all, or almost all of these being invariably impeded or vitiated by the presence of inflammation. The most important secretions are those from the liver, skin, and intestinal canal. "Purgatives afford relief in nearly the same manner as the abstraction of blood from the arm; for a pint of serum will pass off with the fæces, after taking a brisk cathartic." The mere presence of fæces, the secretions being unimpaired, is, comparatively speaking, of very little consequence, as every practitioner must have observed.

"It is of little use to produce action in the intestines, unless you also excite the secretion of the liver; therefore, give mercurials with your saline medicines, as these produce secretion of bile: do not give saline aperients alone, which act chiefly upon the intestines; the best plan is, to administer some mercurial medicine at night, and a purge in the morning." 67.

The following rule of practice of an old Scotch physician is worth recording in this place.

"An old Scotch physician, for whom I had a great respect, and whom I frequently met professionally in the city, used to say, as we were entering the patient's room together, 'Weel, Mister Cooper, we ha' only twa things to keep in meend, and they'll searve us for here and herea'ter; one is always to have the fear of the Laird before our ees, that 'ill do for herea'ter; and the t'other is to keep your booels open, and that will do for here.' 68.

Here Sir Astley lets fall some hints relative to the preservation of his own health, which hints have been retailed in all the popular publications of the day. These rules are simple but rational. Temperance, early rising, sponging the body every morning with cold water, have been pursued for 30 years by the worthy lecturer, and during that time he has hardly ever caught a cold. When indisposed, a dose of calomel and colocynth at night, with some warm diluent before getting up in the morning, carries off the complaint. Long may the able Baronet continue to profit by his code of hygiene! There are some people so tenacious of health, and so amply supplied with restorative powers when exposed to disease, that the very causes of indisposition in others, are to them fortifiers of the constitution. Take, for instance, alternations of atmospheric temperature, the prolific source of illness to so many of the human race. In a well poised constitution these alternations actually strengthen the whole fabric, by action and re-action; while in those who have any weak point about them, especially in the thoracic organs, there is a constant succession of morbid feelings, if not actual disorder. The code therefore which proves salutary to one person may prove deleterious to another. By the means abovementioned, our excellent teacher has been able to go out of the crowded and heated lecture-room into the squares of the hospital, in the severest winter nights, with merely silk stockings on his legs, and yet he has rarely or never caught a cold.

The treatment of chronic inflammation is handled by our author with great skill. He justly observes that the remedies in this disease must have a slow and gradual action on the secretions. "We cannot take this disease by storm; and if our remedies act with violence, we will produce mischief instead of affording relief." "The principle on which this disease is founded, is the arrest of some of the secretions; and its successful treatment depends upon their restoration."

"Chronic inflammation is frequently produced through the influence of the mind on the body. Thus long-continued grief will stop the secretion of the bile; anxiety of mind produces disease in the breasts. But whatever is the cause of the arrest of secretion, some congestion is the result; as enlargement of the liver, of glands, or of joints; the formation of common tumours, or those of a specific character, as fungus or scirrhus.

"In disease of a chronic kind, give calomel and opium; and I cannot point out to you a better example of its good effects than is observable in chronic inflammation of the joints, or testicle; in the former cases, when assisted by counter-irritation, it is by far the best remedy. The most common medicine, and probably, as a general one, the best that is administered in chronic inflammation, is the pilul. hydrarg. submur. comp.: it acts at the same time on the liver, intestines, and skin; and if you can succeed in restoring these, the disease will disappear, and its effects will be absorbed; for, by these medicines, in proportion as you increase the secretions, you excite the action of the absorbent vessels.

"Another excellent medicine, for the cure of chronic complaints, is the oxymuriate of mercury (corrosive sublimate), dissolved in nitrous æther, and combined with tincture of bark or of rhubarb, or with the decoction of sarsaparilla; continue it for some time, watching its effects with care, always keeping in mind that mercury, given to excess, will tend to increase rather than destroy constitutional irritation: as sarsaparilla seems to possess the power of lessening irritability, we frequently give it with mercury, and in this combination they are administered with the greatest advantage.

"Chronic disorders in children require small doses of the hydrarg. e cretâ and rhubarb mixed together, and given every night and morning; this compound is exceedingly mild, and will have a particularly benign influence. In children also, one grain of the oxymuriate of mercury, dissolved in an ounce of tincture of bark, and given in doses of from half a drachm to one drachm, twice a day, in water, according to the age and constitution of the child, is a very valuable medicine. It is said, that the oxymuriate is decomposed by the tincture of bark; but whether it is so or not, it is attended with so many good effects, that I strongly recommend it, particularly in diseases of the mesenteric glands. Calomel and rhubarb, the hydrargyrus e cretâ and soda, are also medicines of much power in the chronic diseases of children.

"Lastly, in some cases, it is not advisable to give these little creatures mercury; a medicine composed of rhubarb and carbonate of iron, or of rhubarb, soda, and calumba, given often, and in small doses, will be more beneficial, as these act as aperients, improve the digestive functions, increase the appetite, and restore the general health, without the danger of exciting irritation at the moment, or rendering the system afterwards irritable." 73.

Sir Astley's directions as to the *local* treatment of inflammation, are, as might be expected, of the most practical and skilful kind. One of the best lotions that can be applied to an inflamed part, he observes, for the purpose of producing cold, is a mixture of one ounce of rectified spirit to five of water. The liq. plumb. acet. dilut. does not seem to be a favourite with the lecturer. He has known its long-continued use produce partial paralysis. In applying the solution above recommended the linen should be fine, and put lightly on the inflamed part, in order that evaporation may go on with facility, this being the grand object in view. He does not recommend the application of ice to an inflamed part. It irritates and is apt to produce gangrene. This advice does not apply to deep-seated inflammations, as of the brain. "Here the application of ice is of signal service." It is, indeed! we have often seen raging mania from inflammation of the membranes of the brain calmed in a few hours, by cold to the shaven crown, and a strong band of leeches round the forehead, temples, and behind the ears.

It is a seeming contradiction (in which articles, by the way, our science abounds) to attempt the relief of inflammation by *heat*. It is like blowing hot and cold with the same breath. The application of heat alone, however, would be injurious, by exciting, not soothing the parts. But when combined with moisture, it is beneficial, by producing relaxation, opening the cutaneous pores, and giving rise to perspiration—thereby removing congestion, and producing effects nearly similar to those which arise from a blister. Our author does not think that medicated fomentations are preferable to those made with warm water, unless the integuments be broken. We are inclined to think with Mr. Tyrrell (in a note on this passage) that anodyne fomentations and poultices are of considerable use, whether the integuments be broken or sound. Sir Astley's directions respecting local bleeding, counter-irritation, and stimulating applications in the treatment of chronic inflammation, are excellent, but cannot, of course, contain any thing novel or peculiar. Position and rest are of great consequence in the methodus medendi, though too often overlooked by young practitioners. The removal of induration left after chronic inflammation is effected by various means, as pressure, electricity, mercury, friction, &c. Each of which means is illustrated by cases from our author's extensive practice.

With this short specimen, we conclude our first notice of these distinguished lectures. We shall not analyse all the lectures; but select three or four occasionally for an article in each of the succeeding Nos. of the Journal, taking an opportunity to mingle with them such observations and reflections, as we may deem necessary. In the mean time we think we need hardly say how well the young practitioner would do to place these lectures in his library, as a never-failing resource for reference and study. Even those who may have purchased the notes of these lectures, published in another form, will find it to their advantage to have the authentic copy of them, corrected by the pen of their admirable author.

